# Tumor Treating Fields: At the Crossroads Between Physics and Biology for Cancer Treatment

**DOI:** 10.3389/fonc.2020.575992

**Published:** 2020-10-30

**Authors:** Francesca A. Carrieri, Caleb Smack, Ismaeel Siddiqui, Lawrence R. Kleinberg, Phuoc T. Tran

**Affiliations:** ^1^Department of Radiation Oncology and Molecular Radiation Sciences, Johns Hopkins University School of Medicine, Baltimore, MD, United States; ^2^Program in Cancer Invasion and Metastasis, The Sidney Kimmel Comprehensive Cancer Center, Johns Hopkins University School of Medicine, Baltimore, MD, United States; ^3^Department of Urology, Johns Hopkins University School of Medicine, Baltimore, MD, United States; ^4^Program in Cellular and Molecular Medicine, Johns Hopkins University School of Medicine, Baltimore, MD, United States

**Keywords:** Tumor Treating Fields, alternating electric fields, mitosis, cancer treatment, TTFields, centrosome, mitotic spindle

## Abstract

Despite extraordinary advances that have been achieved in the last few decades, cancer continues to represent a leading cause of mortality worldwide. Lethal cancer types ultimately become refractory to standard of care approaches; thus, novel effective treatment options are desperately needed. Tumor Treating Fields (TTFields) are an innovative non-invasive regional anti-mitotic treatment modality with minimal systemic toxicity. TTFields are low intensity (1–3 V/cm), intermediate frequency (100–300 kHz) alternating electric fields delivered to cancer cells. In patients, TTFields are applied using FDA-approved transducer arrays, orthogonally positioned on the area surrounding the tumor region, with side effects mostly limited to the skin. The precise molecular mechanism of the anti-tumor effects of TTFields is not well-understood, but preclinical research on TTFields suggests it may act during two phases of mitosis: at metaphase, by disrupting the formation of the mitotic spindle, and at cytokinesis, by dielectrophoretic dislocation of intracellular organelles leading to cell death. This review describes the mechanism of action of TTFields and provides an overview of the most important *in vitro* studies that investigate the disruptive effects of TTFields in different cancer cells, focusing mainly on anti-mitotic roles. Lastly, we summarize completed and ongoing TTFields clinical trials on a variety of solid tumors.

## Introduction

The paradigm of standard care for cancer treatment has dramatically changed in the past two decades due to a greater understanding of tumor development and treatment resistance to more classical therapies (1) including novel immune and molecularly targeted therapies (2). Tumor Treating Fields (TTFields) are a recently developed distinct antineoplastic therapy consisting of low-intensity and intermediate frequency alternating electric fields. Data supporting clinical effectiveness accumulated from preclinical and clinical studies since their initial proposal (3) led to US Food and Drug Administration (FDA) approval as a single agent therapy in recurrent glioblastoma (GBM), as adjuvant therapy along with standard chemoradiation for postoperative glioblastoma (4, 5), and for therapy of mesothelioma (6).

TTFields are applied to the localized tumor using an array of ceramic applicators powered by a portable 3 lb battery pack. These fields are believed to exert their inhibitory effect on dividing cells by inducing a disruption of cytokinesis during mitosis, leading to cell cycle arrest and cell death (7). This review describes the TTFields putative mechanisms of action, recapitulating the key *in vitro* investigations. Finally, we provide an up-to-date summary of approved and ongoing clinical studies highlighting the multifaceted applications of TTFields.

## Tumor Treating Fields: Physics Primer and Putative Mechanisms of Action

### Low Intensity Alternating Electric Fields and Seminal Use as an Anti-mitotic

With a basic understanding of Gauss's law and Coulombic attractions between oppositely charged particles, one can visualize electric fields through a thought experiment involving a parallel plate capacitor connected to a battery. By definition, an electric field produces a voltage difference in space (8). When the battery is on, a uniform electric field is generated with a constant voltage between the two capacitor plates, with an infinite number of parallel field lines of equal strength traveling from the negative to the positive end. However, TTFields create a non-uniform electric field by introducing curvature or by alternating, with intermediate frequency (100–300 kHz) and low-intensity (1–3 V/cm), surface conductivity; the generated electric field lines are no longer uniform, but vary in magnitude, are more concentrated near the charges, and become curved (9). These non-uniform electric fields can affect tumor cell growth and cell division (10, 11); importantly, this effect was observed only in dividing cells, and not quiescent cells. A more extensive description of electric fields and their effects have been elegantly reviewed by Kolosnjaj-Tabia and colleagues (12).

The pioneering study from the Palti group reported for the first time that alternating electric fields affect tumor cells. By using insulated electrodes with an intensity of 2 V/cm and a frequency between 100 and 300 kHz, they observed cell cycle arrest and cell rupture, leading to cell death in different actively dividing tumor cells, *in vitro*. Similarly, these effects were observed *in vivo* in two different animal models, resulting in a significant tumor growth reduction (3). These data not only prove the effects of TTFields on biological processes both *in vitro* and *in vivo*, but importantly, paved the way for the development of a novel therapeutic intervention for different cancer types. These early developments led to more detailed molecular explanations through which TTFields exert their function on dividing cells.

### TTFields and Mechanisms for Anti-mitotic Effects

Preclinical studies to date suggest TTFields have two major effects on cancer cells, namely prolonged mitosis and mitotic spindle assembly disruption and cell membrane destruction close to the cleavage furrow during telophase. The established mechanisms through which TTFields are believed to exert effects on dividing cells include: (1) impairment of mitotic spindle microtubule formation; and, (2) the dielectrophoretic effect, in telophase/cytokinesis, which compromises organelles and biomolecules impairing chromosomal segregation and cell division. These mechanisms have been suggested to result in cancer cell death, offering a therapeutic effect by reducing tumor growth.

The cell cycle is an essential process consisting of four phases, G1, S, G2, and M linked to cell division resulting in two daughter cells. The first three phases, known as interphase, are associated with cellular growth and genetic material duplication, ultimately segregated to daughters. Mitosis (M), is a multi-step process, orchestrating dense DNA compaction into chromosomes that are evenly distributed among daughters, culminating in their separation from the parent cell. The macromolecular machine segregating chromosomes among the two daughter cells during mitosis is known as the mitotic spindle. Its main components, the microtubules, are formed from α- and β-tubulin dimers. Microtubules are in a dynamic state of polymerization/depolymerization which is important for the cytoskeleton remodeling during mitosis (13–15).

**Figure 1:**
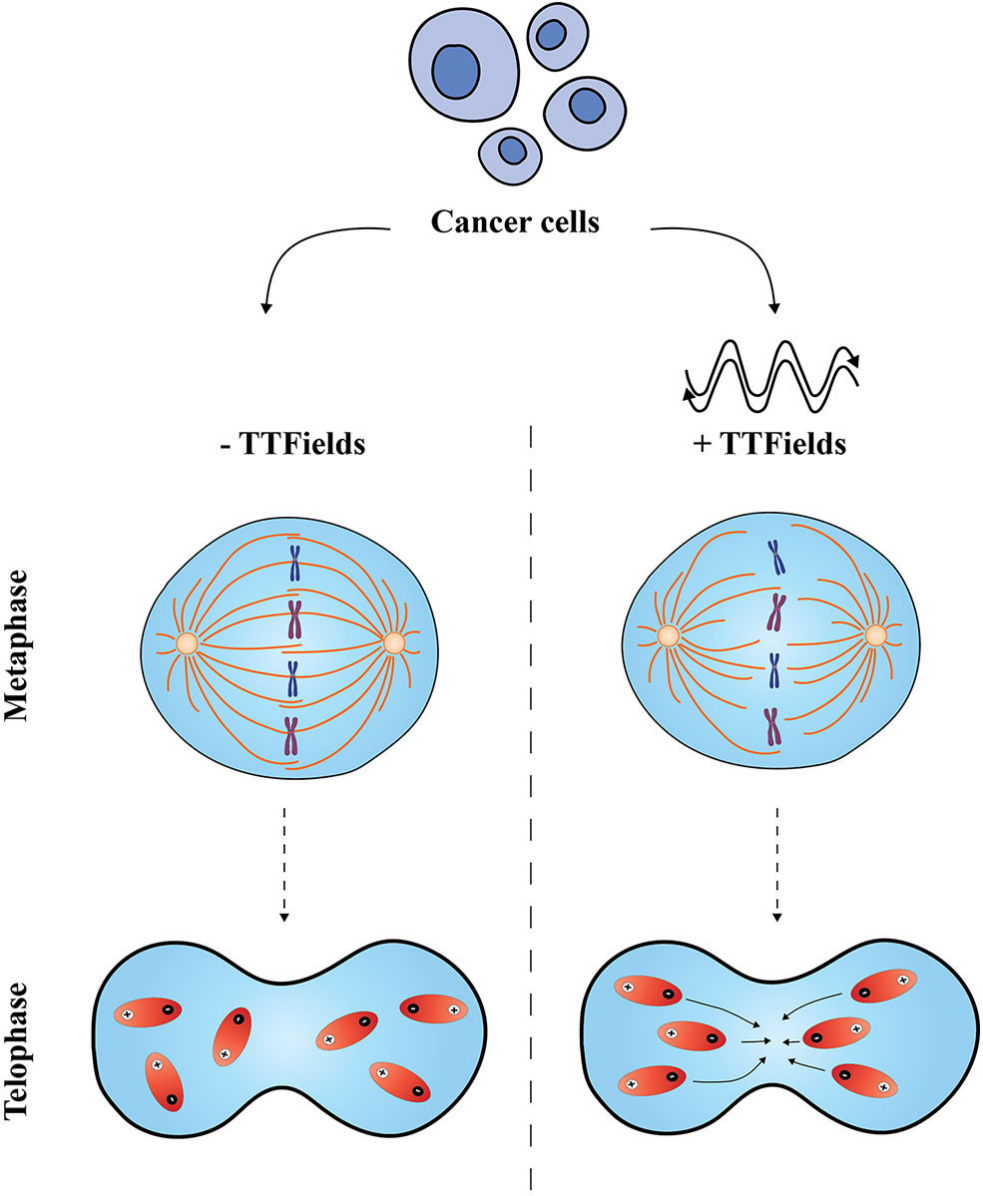
Proposed effects of Tumor Treating Fields on cancer cells. The application of TTFields is believed to disrupt two distinct phases in the mitotic process. In metaphase, TTFields interfere with the mitotic spindle assembly and disturb the alignment of tubulin subunits; in telophase, non-uniform electric fields cause a change in the cell shape and conformation, and compromise polar elements (shown in red) which move to the cleavage furrow leading to an impaired cell division (right-hand side). Normal metaphase and telophase are shown on the left-hand side.

Much of the focus of TTFields mechanism of action lies in mitosis (Figure 1). This anti-mitotic effect has been investigated by Giladi et al., in different cancer cells, exposing them to TTFields. Under these conditions, actively dividing treated cells displayed a decreased ratio between polymerized and total tubulin, resulting in an impaired mitotic spindle organization. Furthermore, upon treatment with TTFields, cancer cells displayed altered chromosomal count, suggesting that TTFields induce cell aneuploidy. The disruption of the mitotic spindle machinery, critical for mitosis, results in abnormal chromosome formation and formation of multinucleated cells. Giladi et al. demonstrated that TTFields exposure to cells *in vitro* phenocopied defects in the mitotic spindle and in some cases caused cells to undergo mitotic catastrophe or a form of cell death-linked to grossly abnormal mitosis. Further analyses of TTFields-treated tumor cells showed disruptive mitotic spindle formation *in vivo* and a prolonged mitosis. Detailed cell cycle analysis of exposed cells revealed that there was significant accumulation of cells in M phase (compared to untreated cells) and the majority of treated cells underwent a caspase-mediated apoptosis (16).

The second proposed mechanism by which alternating electric fields affect cell division is by dielectrophoresis, a phenomenon occurring in neutral particles where their motion is induced by a non-uniform electric field between electrodes (17). In this context, TTFields have been proposed to impair cell division by dielectrophoresis of the mitotic cleavage furrow during telophase. TTFields potentially affect polar biomolecules by moving them close to the furrow region, leading to a defective telophase/cytokinesis (18). A recent mathematical modeling investigated the correlation between electric forces and mitosis, showing that cells with a narrow mitotic furrow during telophase/cytokinesis were more sensitive to TTFields, compared to the control. At this narrow mitotic region, the power absorption (as consequence of TTFields delivery) was higher and dependent on field frequency. This effect was only appreciated when the mitotic furrow was aligned and parallel to the electric field and may explain why cell proliferation is not completely blocked by TTFields, but only partially decreased (19). Subsequent computational modeling calculated the electric field distribution in the brain during TTFields therapy and investigated the reliability of their predictions with respect to heterogeneous, anisotropic dielectric properties. Using virtual head models, TTFields treatment on brain tumors *in silico* was successfully replicated (20).

Korshoej et al. estimated the required anti-tumor dose and optimized a treatment plan of TTFields for cancer therapy. This study offers a new perspective on TTFields therapy considering complex conductivity distributions of the head and addressing individual differences in patient anatomy and tumor morphology previously unconsidered. The authors described the principal component decomposition of average field vectors induced over an activation cycle, which quantifies both the mean intensity and unwanted spatial correlation of TTFields, with the hope of combining the two values into a single measure of clinical significance (21, 22).

Lastly, research investigated the novel anti-tumor effect of altering the tumor cell membrane potential by targeting ion channels *via* TTFields induced dielectrophoresis. Theoretical calculations postulate that the electromagnetic forces generated by TTFields alone are too weak relative to Brownian motion to have a significant effect on tubulin dimers alignment and hence cannot have a direct mechanical effect on the cytoskeleton during the early stages of mitosis. TTFields instead were hypothesized to generate changes in the cell membrane potential that initiate apoptosis within dividing tumor cells while sparing non-dividing cells. The TTFields-induced membrane potential change across the cell membrane of normal cells is only about 3% of the non-dividing cell membrane potential, while in the dividing tumor cell membrane potential can be as high as 17%. Mechanistically, as Ca^2+^ is a regulator of microtubule polymerization, manipulating the calcium ion channel may offer a mechanistic clue to an anti-tumor effect. Altering the cell membrane potential of a tumor cell in prophase and increasing Ca^2+^ flow into the cell could decrease microtubule polymerization. The disruption of ionic homeostasis thus offers a unique explanation regarding the ability of TTFields to exhibit an anti-microtubule effect during the early stages of mitosis (23).

Centrosomes are critical organelle structures serving as the major cell microtubule organizing center, critical for animal cell mitosis, and present in one copy until the time leading up to cell division where one duplication is made. The presence of supernumerary centrosomes (SNC) in the majority of tumors has been observed and believed to contribute to genomic instability through chromosome mis-segregation errors (24). Many cancers acquire this form of genomic instability by eroding the pathways that serve to maintain genome integrity (25). Cancers with SNC seem to increase the frequency of chromosome segregation errors, but at the same time in order to survive and overcome mitotic catastrophe, cancer cells cluster their SNC into two spindle poles, a phenomenon known as centrosome clustering (26). Centrosome clustering inhibition may provide a means to selectively kill cancer cells with SNCs, forcing them into lethal multipolar divisions without affecting cell division of normal cells without SNC. Further research on centrosomes could reveal whether TTFields exert their function selectively on tumor cells in general by affecting microtubule biology and specifically on cells with SNC.

### TTFields and Non-mitotic Cellular Effects

Over the years, several *in vitro* studies conducted on human cancer cell lines have reported the non-mitotic effects of TTFields (3, 16, 27). A set of more recent studies have shown that TTFields could render cancer cells less efficient in their DNA damage repair capacity, with cells showing higher DNA damage and replication stress (28, 29). Gene expression analysis on a variety of non-small cell lung cancer (NSCLC) cell lines treated with TTFields revealed not only changes in cell cycle and mitosis-related pathways, but also in DNA damage response pathways. Results from ingenuity pathway analyses, confirmed by immunoblotting in four different cancer cell lines, revealed, upon exposure to TTFields, a significant down-regulation of BRCA1, a well-known tumor suppressor involved in DNA double breaks repair and maintaining genomic stability through cell cycle checkpoints. In addition, Story and colleagues demonstrated that TTFields induce DNA double strand breaks, as confirmed by the formation of γ-H2AX foci and reduce DNA damage repair following radiation. Interestingly, TTFields enhanced cancer cell sensitivity to radiation when cells where exposed to radiation after TTFields delivery, opening up new possibilities for developing novel radiosensitization treatment protocols. Taken together, these data proposed an additional mechanism of action of TTFields *in vitro* (28, 29). Although the biological effects of TTFields have been explored, the full knowledge regarding TTFields biophysical mechanism of action against cancer cells is still likely an area ripe for further investigation.

## TTFields as a Cancer Therapy

In the following section we review application of TTFields as a cancer therapy both in preclinical cancer mouse models and in human clinical trials as monotherapy and in combination treatment trials for different solid tumors.

### Preclinical Studies in Mouse Models

In some of the first *in vivo* evidence for the TTFields effect on cancer cells, mice intradermally inoculated with malignant melanoma (B16F1) or adenocarcinoma cells (CT-26) and TTFields-treated exhibit a significant tumor growth inhibition compared to non-treated tumors (3). Similar results were observed in rats intracranially inoculated with F-98 glioma cells and treated with TTFields (11) as well as in B16F10 melanoma cells injected into mice. After TTFields delivery, tumor volume reduction, and prolonged animal lifespan were reported, compared to control. Increased apoptosis, reduced CD34-positive cells and decreased level of VEGF protein were also shown. These findings propose a potential mechanism associated with TTFields, by disturbing tumor blood vessels and consequently leading to tumor growth inhibition (30). Further investigations have suggested that TTFields exert their effects on tumor blood vessels by downregulating VEGF and/or HIF-1α suppressing angiogenesis (31).

TTFields also inhibit solid tumor metastases in mice with malignant melanoma and rabbits with squamous cell carcinoma. In both cases, not only did treated animals showed a longer lifespan, but a lower number of lung metastasis was observed compared to the control. Although a mechanistic explanation was not provided, the authors speculated that TTFields could enhance the immune response to cancer cells or directly inhibit migration and invasion of tumor cells leading therefore to lower metastases (32).

Lastly, preclinical studies have also reported combination treatments that are enhanced with TTFields. The efficacy of TTFields combined with pemetrexed, cisplatin, or paclitaxel in NSCLC, was evaluated demonstrating that tumor growth inhibition was superior compared to the single arm treatments (33). Similarly, enhanced anti-tumor effects of combined TTFields-paclitaxel was demonstrated in ovarian cancer (34).

### Clinical Trials

#### Recurrent Glioblastoma as a Single Agent

The first trial ever conducted using TTFields as monotherapy was against GBM initiated in 2007 on a small cohort of 10 patients (11). The safety results from this study led to a phase II trial where TTFields was delivered after radiotherapy and adjuvant temozolomide (TMZ) showing a median overall survival (OS) >39 months (35). A randomized trial (EF-11) included 120 GBM patients treated with TTFields showed similar OS and response compared to 117 patients who received standard systemic therapies (36). Based on this data, in 2011, the FDA approved the first-generation TTFields device (NovoTTF-100A) as therapy for recurrent GBM (4).

#### Newly Diagnosed Glioblastoma Along With Standard Therapy

The multicenter, open-label, randomized phase III EF-14 trial enrolled 695 patients with newly diagnosed GBM to assess the TTFields effectiveness when administered after completion of concurrent chemoradiation. The trial positive results led FDA approval of an improved TTFields device (Optune), for newly diagnosed GBM patients (5). The trial randomized patients to receive TTFields after completion of chemoradiation in combination with standard adjuvant TMZ or TMZ as single treatment. Median progression-free survival from randomization was 6.7 vs. 4.0 months (*P* < 0.001) and median OS was 20.9 vs. 16.0 months (*P* < 0.001), both in favor of the TTFields-TMZ combination. Systemic and neurologic adverse events and health-related life quality were similar, but mild-to-moderate skin toxicity underneath the transducer arrays occurred in 52% of patients who received TTFields-TMZ (37–40). The planned TRIDENT trial will randomize patients to standard TTFields initiated during adjuvant TMZ against earlier initiation during concurrent chemoradiation and continuing through adjuvant TMZ.

#### Mesothelioma

A chest applicator NovoTTF-100L has been developed to administer TTFields to the thoracic cavity. The STELLAR trial resulted in a median OS of 18.2 months (compared to 12.1 months in historical controls) for patients treated with TTFields with inoperable and previously untreated mesothelioma in combination with pemetrexed and cisplatin or carboplatin (6). FDA approval of NovoTTF-100L was granted in 2019 for use along with pemetrexed and platinum-based chemotherapy for first-line treatment of unresectable, locally advanced or metastatic, malignant pleural mesothelioma.

#### Other Solid Tumors

Additional clinical trials in other histologies have also evaluated the efficacy of TTFields. In the LUNAR trial, unresectable advanced NSCLC were treated with TTFields in combination with pemetrexed and showed treatment tolerability with no adverse reactions (expect localized mild dermatitis), and 1- and 2-years survival of 53 and 27%, respectively (41). Based on these promising results, a LUNAR phase III randomized trial is currently ongoing. The study will evaluate the TTFields treatment efficacy with docetaxel or anti-PD1 in NSCLC (42).

TTFields treatment against other solid malignancies, such as ovarian cancer and pancreatic adenocarcinoma, were examined in INNOVATE and PANOVA phase II clinical trials, respectively. The INNOVATE was a single arm trial testing the efficacy of TTFields combined with paclitaxel in patients with recurrent ovarian carcinoma. The 6-months and 1- year survival rates were 90 and 61%, respectively (43). The PANOVA study involved forty patients with newly diagnosed, locally advanced, or metastatic pancreatic ductal adenocarcinoma (PDAC). Patients received combination of TTFields and gemcitabine (with a median progression-free survival of 8.3 months) or gemcitabine and nab-paclitaxel (with a median progression-free survival of 12.7 months). No adverse events were reported, and the treatment was tolerable (44). A phase III PANOVA-3 trial is currently ongoing (45). Several clinical trials investigating TTFields are underway and listed with completed clinical trials in Table 1.

**Table 1:** Summary of completed and ongoing clinical trials using TTFields as monotherapy or in combination with other therapeutic agents to treat solid malignancies.

**Completed Clinical Trials**					
**Clinical trial name**	**Phase**	**Cancer type**	**Treatment modality**	**Results**	**References**
EF-11	III	Recurrent GBM	TTFields vs. chemotherapy	OS of 6.6 months	(36)
EF-14	III	GBM	TTFields + temozolomide vs. temozolomide	OS of 20.5 months	(37)
LUNAR	II	Unresectable advanced NSCLC	TTFields + pemetrexed	OS of 13.4 months	(41)
INNOVATE	II	Recurrent ovarian carcinoma	TTFields + paclitaxel	PFS of 8.9 months	(43)
PANOVA	II	Locally advanced or metastatic PDAC	TTFields + gemcitabine and TTFields + gemcitabine + nab-paclitaxel	PFS of 8.3 months and PFS of 12.7 months	(44)
STELLAR	II	Unresectable and previously untreated mesothelioma	TTFields + pemetrexed + cisplatin or carboplatin	OS of 18.2 months	(6)
**Ongoing Clinical Trials**					
INNOVATE-3	III	Recurrent ovarian cancer	TTFields + Paclitaxel	N/A	https://ClinicalTrials.gov/show/NCT03940196
PANOVA-3	III	Locally advanced PDAC	TTFields + Gemcitabine and TTFields + gemcitabine + nab-paclitaxel	N/A	https://ClinicalTrials.gov/show/NCT03377491
HEPANOVA	II	Advanced hepatocellular carcinoma	TTFields + Sorafenib	N/A	https://ClinicalTrials.gov/show/NCT03606590
LUNAR	III	Stage IV NSCLC	TTFields + anti-PD-1 or docetaxel	N/A	https://ClinicalTrials.gov/show/NCT02973789
METIS	III	1–10 brain metastases from NSCLC	TTFields following radiosurgery	N/A	https://ClinicalTrials.gov/show/NCT02831959
COMET	II	1–5 brain metastases from NSCLC	TTFields following optimal standard local treatment	N/A	https://ClinicalTrials.gov/show/NCT01755624
2-THE-TOP	II	Newly diagnosed GBM	Adjuvant TTFields + temozolomide and pembrolizumab	N/A	https://Clinicaltrials.gov/ct2/show/NCT03405792
N/A	I/II	Newly diagnosed GBM	TTFields + temozolomide + radiation therapy	N/A	https://ClinicalTrials.gov/show/NCT03705351 https://ClinicalTrials.gov/show/NCT03477110
N/A	II	Unresectable gastric adenocarcinoma	TTFields + oxaliplatin or capecitabine or trastuzumab	N/A	https://ClinicalTrials.gov/show/NCT04281576
N/A	II	Brain metastases from SCLC	TTFields following stereotactic radiosurgery	N/A	https://ClinicalTrials.gov/show/NCT03488472
N/A	II	Recurrent GBM	TTFields + nivolumab +/- ipilimumab	N/A	https://ClinicalTrials.gov/show/NCT03430791
TIGER	N/A	GBM	TTFields	N/A	https://ClinicalTrials.gov/show/NCT03258021
N/A	II	Brain metastases from SCLC	TTFields	N/A	https://ClinicalTrials.gov/show/NCT03995667
N/A	II	Recurrent GBM	TTFields + niraparib	N/A	https://Clinicaltrials.gov/show/NCT04221503

*TTFields, Tumor Treating Fields; GBM, glioblastoma multiforme; NSCLC, non-small cell lung cancer; PDAC, pancreatic ductal adenocarcinoma; SCLC, small cell lung cancer; OS, overall survival; PFS, progression-free survival; N/A, not available*.

## Conclusions

The movement from initial laboratory observations to the implementation of several clinical trials have shown the significant strides TTFields have made as a promising therapeutic agent for the treatment of different solid cancers. Likely through the disruption of cell proliferation and tumor growth by curtailing mitotic activity, TTFields show promise as an innovative, non-invasive anti-cancer treatment modality. However, there remain questions regarding exactly how TTFields exert anti-cancer effects on cancer cells. Mathematical modeling suggests TTFields do not produce strong anti-mitotic effects through microtubule disruption but instead induce changes in cell membrane potential causing deleterious downstream effects during cell separation at the cleavage furrow. Additionally, several other open-ended mechanistic questions remain to be answered. The action of TTFields in each phase of the cell cycle and the link between TTFields, DNA repair pathways and mitotic catastrophe remain to be characterized. One promising area of investigation could evaluate whether TTFields exert their function selectively on tumor cells by affecting microtubule biology through centrosomes. Surely, gaining more insights into TTFields mechanisms will pave the way for further innovative clinical trials. With six completed clinical trials to date, and 14 ongoing at different phases, a deeper understanding of TTFields biology is crucial to identify novel vulnerabilities in specific malignancies that might be efficiently targeted by TTFields, either as monotherapy or in combination with other therapeutics.

## Author Contributions

FC and PT conceived the structure and content. FC wrote the initial draft document, designed, and produced the figures. CS, IS, and LK reviewed the document. PT corrected and edited the document. All authors contributed to the article and approved the submitted version.

## Conflict of Interest

FC has received research funding from Novocure for *in vitro* studies investigating the efficacy of TTFields and inhibition of centrosome clustering. PT has served as a consultant for RefleXion Medical, Inc., Noxopharm, and Janssen-Taris Biomedical and has research grant support from RefleXion Medical, Inc., Astellas Pharmaceuticals, and Bayer Healthcare. The remaining authors declare that the research was conducted in the absence of any commercial or financial relationships that could be construed as a potential conflict of interest.
